# 5-(4-Chloro­phen­yl)-6-isopropyl-5,6-dihydro-4*H*-pyrrolo­[3,4-*c*]isoxazole

**DOI:** 10.1107/S1600536810034872

**Published:** 2010-09-04

**Authors:** Kwang Ha, Hyun Sub Lim, Hyung Jin Kim

**Affiliations:** a School of Applied Chemical Engineering, Chonnam National University, Gwangju 500-757, Republic of Korea

## Abstract

The title compound, C_14_H_15_ClN_2_O, contains an eight-membered 5,5-fused bicycle with two substituents. The dihedral angle between the nearly planar eight-membered ring [maximum deviation = 0.033 (2) Å] and the benzene ring is 25.0 (1)°. In the crystal structure, mol­ecules are stacked in columns along the *b* axis and C—H⋯π inter­actions are observed between the columns.

## Related literature

For the synthesis of the title compound, see: Kim & Lee (1994 ▶). For the biological activity of isoxazoles, see: Boyd (1991 ▶); Kim *et al.* (1994 ▶, 1997 ▶, 1999 ▶); Lang & Lin (1984 ▶); Sutharchanadevi & Murugan (1996 ▶).
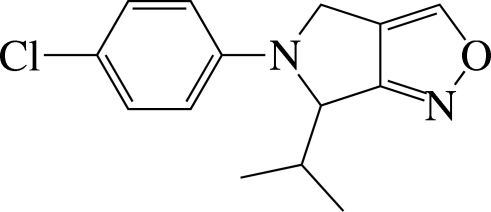


## Experimental

### 

#### Crystal data

C_14_H_15_ClN_2_O
*M* *_r_* = 262.73
Monoclinic, 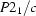

*a* = 15.0037 (9) Å
*b* = 6.2364 (4) Å
*c* = 15.5801 (9) Å
β = 117.238 (1)°
*V* = 1296.16 (14) Å^3^
*Z* = 4
Mo *K*α radiation
μ = 0.28 mm^−1^
*T* = 200 K
0.35 × 0.28 × 0.12 mm

#### Data collection

Bruker SMART 1000 CCD diffractometer
Absorption correction: multi-scan (*SADABS*; Bruker, 2000 ▶) *T*_min_ = 0.861, *T*_max_ = 0.966
9224 measured reflections
3211 independent reflections
1907 reflections with *I* > 2σ(*I*)
*R*_int_ = 0.043

#### Refinement

*R*[*F* ^2^ > 2σ(*F* ^2^)] = 0.044
*wR*(*F* ^2^) = 0.117
*S* = 1.07
3211 reflections
165 parameters
H-atom parameters constrained
Δρ_max_ = 0.36 e Å^−3^
Δρ_min_ = −0.44 e Å^−3^

### 

Data collection: *SMART* (Bruker, 2000 ▶); cell refinement: *SAINT* (Bruker, 2000 ▶); data reduction: *SAINT*; program(s) used to solve structure: *SHELXS97* (Sheldrick, 2008 ▶); program(s) used to refine structure: *SHELXL97* (Sheldrick, 2008 ▶); molecular graphics: *ORTEP-3* (Farrugia, 1997 ▶) and *PLATON* (Spek, 2009 ▶); software used to prepare material for publication: *SHELXL97*.

## Supplementary Material

Crystal structure: contains datablocks global, I. DOI: 10.1107/S1600536810034872/is2594sup1.cif

Structure factors: contains datablocks I. DOI: 10.1107/S1600536810034872/is2594Isup2.hkl

Additional supplementary materials:  crystallographic information; 3D view; checkCIF report

## Figures and Tables

**Table 1:** Hydrogen-bond geometry (Å, °) *Cg*1 is the centroid of the C1–C6 ring.

*D*—H⋯*A*	*D*—H	H⋯*A*	*D*⋯*A*	*D*—H⋯*A*
C5—H5⋯*Cg*1^i^	0.95	2.65	3.405 (3)	136
C9—H9⋯*Cg*1^ii^	0.95	2.62	3.392 (3)	138

Symmetry codes: (i) 
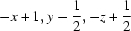
; (ii) 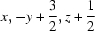.

**Fig. 1.**
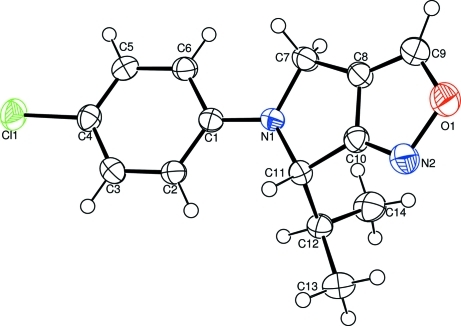
The structure of the title compound, with displacement ellipsoids drawn at the 50% probability level for non-H atoms.

**Fig. 2.**
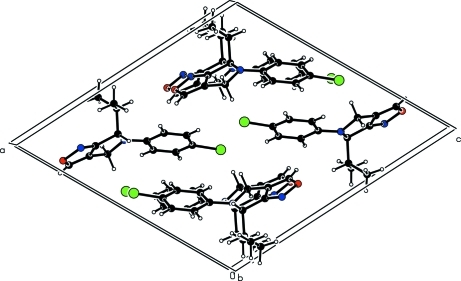
View of the unit-cell contents of the title compound.

## supplementary crystallographic information

### Comment

Isoxazole derivatives bearing various substituents are known to have diverse biological activities in pharmaceutical and agricultural areas (Lang & Lin, 1984; Boyd, 1991). Dihydropyrrolo[3,4-*c*]isoxazole, a fused bicyclic isoxazole, is interesting to develop a new lead compound as a plant fungicide because bicyclic isoxazoles such as dihydrofuro[3,4-*c*]isoxazole and dihydropyrano[3,4-*c*]isoxazole derivatives particularly have fungicidal activities against some plant pathogens (Kim *et al.*, 1994, 1997, 1999). The title compound was prepared by the known method (Kim & Lee, 1994) with a minor modification.

The title compound, C_14_H_15_ClN_2_O, is an 8-membered 5,5-fused bicycle with two substituents (Fig. 1). The dihedral angle between the nearly planar 8-membered ring [maximum deviation of 0.033 (2) Å for C7] and the benzene ring [maximum deviation of 0.023 (2) Å for C1] is 25.0 (1)°. In the crystal structure, the molecules are stacked in columns along the *b* axis (Fig. 2), and display C—H···*Cg*1 (the centroid of ring C1–C6) interactions (Table 1).

### Experimental

A mixture of 3-methyl-1-nitrobutan-2-yl acetate (1.23 g, 7 mmol), 4-chloro-*N*-(prop-2-ynyl)aniline (3.48 g, 21 mmol) and K_2_CO_3_ (1.16 g, 8.4 mmol) in THF (20 ml) was stirred for 12 h at 25 °C. The mixture was concentrated *in vacuo* and column chromatographed (SiO_2_) by eluting with a mixture of n-hexane/EtOAc (5:1) to afford 4-chloro-*N*-(3-methyl-1-nitrobutan-2-yl)-*N*-(prop-2-ynyl)aniline (0.49 g, 25%). The title compound was prepared by the intramolecular nitrile oxide-alkyne cycloaddition of 4-chloro-*N*-(3-methyl-1-nitrobutan-2-yl)-*N*-(prop-2-ynyl)aniline in the presence of 4-chlorophenylisocyanate and triethylamine (Kim & Lee, 1994). Crystals suitable for X-ray analysis were obtained by slow evaporation from an n-hexane/CHCl_3_ solution.

### Refinement

H atoms were positioned geometrically and allowed to ride on their respective parent atoms [C—H = 0.95 (CH, *sp*^2^), 1.00 (CH, *sp*^3^), 0.99 (CH_2_) or 0.98 Å (CH_3_), and *U*_iso_(H) = 1.2*U*_eq_(C) or 1.5*U*_eq_(methyl C)].

### Crystal data

**Table:**

C_14_H_15_ClN_2_O	*F*(000) = 552
*M**_r_* = 262.73	*D*_x_ = 1.346 Mg m^−^^3^
Monoclinic, *P*2_1_/*c*	Mo *K*α radiation, λ = 0.71073 Å
Hall symbol: -P 2ybc	Cell parameters from 2659 reflections
*a* = 15.0037 (9) Å	θ = 2.6–27.1°
*b* = 6.2364 (4) Å	µ = 0.28 mm^−^^1^
*c* = 15.5801 (9) Å	*T* = 200 K
β = 117.238 (1)°	Stick, pale yellow
*V* = 1296.16 (14) Å^3^	0.35 × 0.28 × 0.12 mm
*Z* = 4	

### Data collection

**Table:**

Bruker SMART 1000 CCD diffractometer	3211 independent reflections
Radiation source: fine-focus sealed tube	1907 reflections with *I* > 2σ(*I*)
graphite	*R*_int_ = 0.043
φ and ω scans	θ_max_ = 28.3°, θ_min_ = 2.6°
Absorption correction: multi-scan (*SADABS*; Bruker, 2000)	*h* = −19→20
*T*_min_ = 0.861, *T*_max_ = 0.966	*k* = −8→8
9224 measured reflections	*l* = −18→20

### Refinement

**Table:**

Refinement on *F*^2^	Primary atom site location: structure-invariant direct methods
Least-squares matrix: full	Secondary atom site location: difference Fourier map
*R*[*F*^2^ > 2σ(*F*^2^)] = 0.044	Hydrogen site location: inferred from neighbouring sites
*wR*(*F*^2^) = 0.117	H-atom parameters constrained
*S* = 1.07	*w* = 1/[σ^2^(*F*_o_^2^) + (0.0311*P*)^2^ + 0.6269*P*] where *P* = (*F*_o_^2^ + 2*F*_c_^2^)/3
3211 reflections	(Δ/σ)_max_ = 0.001
165 parameters	Δρ_max_ = 0.36 e Å^−^^3^
0 restraints	Δρ_min_ = −0.44 e Å^−^^3^

### Special details

**Table:**

Geometry. All e.s.d.'s (except the e.s.d. in the dihedral angle between two l.s. planes) are estimated using the full covariance matrix. The cell e.s.d.'s are taken into account individually in the estimation of e.s.d.'s in distances, angles and torsion angles; correlations between e.s.d.'s in cell parameters are only used when they are defined by crystal symmetry. An approximate (isotropic) treatment of cell e.s.d.'s is used for estimating e.s.d.'s involving l.s. planes.
Refinement. Refinement of *F*^2^ against ALL reflections. The weighted *R*-factor *wR* and goodness of fit *S* are based on *F*^2^, conventional *R*-factors *R* are based on *F*, with *F* set to zero for negative *F*^2^. The threshold expression of *F*^2^ > σ(*F*^2^) is used only for calculating *R*-factors(gt) *etc*. and is not relevant to the choice of reflections for refinement. *R*-factors based on *F*^2^ are statistically about twice as large as those based on *F*, and *R*- factors based on ALL data will be even larger.

### Fractional atomic coordinates and isotropic or equivalent isotropic displacement parameters (Å^2^)

**Table:**

	*x*	*y*	*z*	*U*_iso_*/*U*_eq_	
Cl1	0.53029 (5)	0.72970 (11)	0.07428 (5)	0.0460 (2)	
O1	0.17957 (13)	1.1528 (3)	0.45555 (12)	0.0468 (5)	
N1	0.25711 (13)	0.8986 (3)	0.24578 (13)	0.0292 (4)	
N2	0.17208 (15)	1.2612 (3)	0.37156 (14)	0.0400 (5)	
C1	0.31613 (16)	0.8552 (4)	0.20076 (15)	0.0284 (5)	
C2	0.32447 (17)	1.0003 (4)	0.13595 (16)	0.0317 (5)	
H2	0.2857	1.1280	0.1193	0.038*	
C3	0.38835 (17)	0.9599 (4)	0.09593 (16)	0.0341 (5)	
H3	0.3940	1.0608	0.0530	0.041*	
C4	0.44355 (17)	0.7739 (4)	0.11838 (16)	0.0327 (5)	
C5	0.43342 (16)	0.6228 (4)	0.17806 (16)	0.0333 (5)	
H5	0.4696	0.4918	0.1911	0.040*	
C6	0.37017 (16)	0.6630 (4)	0.21896 (16)	0.0316 (5)	
H6	0.3634	0.5586	0.2600	0.038*	
C7	0.27190 (18)	0.7731 (4)	0.33222 (17)	0.0341 (5)	
H7A	0.3437	0.7411	0.3738	0.041*	
H7B	0.2335	0.6372	0.3140	0.041*	
C8	0.23192 (17)	0.9227 (4)	0.38036 (16)	0.0322 (5)	
C9	0.21539 (18)	0.9522 (4)	0.45747 (17)	0.0412 (6)	
H9	0.2271	0.8481	0.5060	0.049*	
C10	0.20329 (16)	1.1175 (4)	0.33080 (16)	0.0298 (5)	
C11	0.21313 (16)	1.1144 (4)	0.23924 (15)	0.0279 (5)	
H11	0.2625	1.2261	0.2426	0.034*	
C12	0.11400 (17)	1.1478 (4)	0.14745 (16)	0.0325 (5)	
H12	0.1255	1.1103	0.0909	0.039*	
C13	0.0833 (2)	1.3827 (4)	0.13769 (19)	0.0464 (7)	
H13A	0.0713	1.4239	0.1922	0.070*	
H13B	0.0218	1.4036	0.0772	0.070*	
H13C	0.1369	1.4716	0.1373	0.070*	
C14	0.03096 (18)	1.0035 (4)	0.14450 (19)	0.0488 (7)	
H14A	0.0132	1.0470	0.1951	0.073*	
H14B	0.0542	0.8544	0.1551	0.073*	
H14C	−0.0280	1.0161	0.0813	0.073*	

### Atomic displacement parameters (Å^2^)

**Table:**

	*U*^11^	*U*^22^	*U*^33^	*U*^12^	*U*^13^	*U*^23^
Cl1	0.0449 (4)	0.0560 (4)	0.0462 (4)	0.0075 (3)	0.0287 (3)	−0.0022 (3)
O1	0.0517 (11)	0.0585 (12)	0.0343 (10)	0.0084 (9)	0.0233 (9)	−0.0015 (9)
N1	0.0326 (10)	0.0275 (10)	0.0298 (10)	0.0036 (8)	0.0164 (8)	0.0050 (8)
N2	0.0474 (12)	0.0423 (12)	0.0348 (11)	0.0057 (10)	0.0226 (10)	0.0010 (10)
C1	0.0285 (12)	0.0294 (12)	0.0256 (12)	−0.0007 (10)	0.0109 (9)	−0.0007 (9)
C2	0.0340 (13)	0.0306 (12)	0.0314 (13)	0.0048 (10)	0.0158 (10)	0.0041 (10)
C3	0.0370 (13)	0.0362 (14)	0.0310 (13)	−0.0029 (11)	0.0172 (11)	0.0018 (10)
C4	0.0322 (12)	0.0364 (13)	0.0315 (13)	0.0016 (11)	0.0163 (10)	−0.0042 (10)
C5	0.0312 (12)	0.0306 (13)	0.0340 (13)	0.0033 (10)	0.0115 (10)	−0.0008 (10)
C6	0.0300 (12)	0.0314 (12)	0.0308 (12)	0.0003 (10)	0.0117 (10)	0.0027 (10)
C7	0.0414 (13)	0.0312 (13)	0.0331 (13)	0.0030 (11)	0.0199 (11)	0.0064 (10)
C8	0.0317 (12)	0.0369 (13)	0.0291 (12)	−0.0003 (11)	0.0148 (10)	0.0034 (10)
C9	0.0400 (14)	0.0509 (16)	0.0330 (14)	0.0066 (12)	0.0171 (11)	0.0067 (12)
C10	0.0264 (11)	0.0332 (13)	0.0294 (12)	−0.0006 (10)	0.0124 (10)	−0.0002 (10)
C11	0.0275 (11)	0.0290 (12)	0.0286 (12)	−0.0002 (10)	0.0139 (9)	0.0017 (9)
C12	0.0322 (13)	0.0359 (13)	0.0283 (12)	0.0036 (10)	0.0128 (10)	0.0030 (10)
C13	0.0456 (15)	0.0455 (16)	0.0449 (16)	0.0111 (13)	0.0180 (13)	0.0129 (13)
C14	0.0350 (14)	0.0522 (17)	0.0492 (16)	−0.0046 (13)	0.0106 (12)	0.0032 (13)

### Geometric parameters (Å, °)

**Table:**

Cl1—C4	1.749 (2)	C7—H7A	0.9900
O1—C9	1.356 (3)	C7—H7B	0.9900
O1—N2	1.431 (2)	C8—C9	1.346 (3)
N1—C1	1.385 (3)	C8—C10	1.398 (3)
N1—C11	1.482 (3)	C9—H9	0.9500
N1—C7	1.484 (3)	C10—C11	1.500 (3)
N2—C10	1.303 (3)	C11—C12	1.534 (3)
C1—C6	1.401 (3)	C11—H11	1.0000
C1—C2	1.403 (3)	C12—C14	1.521 (3)
C2—C3	1.385 (3)	C12—C13	1.522 (3)
C2—H2	0.9500	C12—H12	1.0000
C3—C4	1.374 (3)	C13—H13A	0.9800
C3—H3	0.9500	C13—H13B	0.9800
C4—C5	1.380 (3)	C13—H13C	0.9800
C5—C6	1.387 (3)	C14—H14A	0.9800
C5—H5	0.9500	C14—H14B	0.9800
C6—H6	0.9500	C14—H14C	0.9800
C7—C8	1.485 (3)		
			
C9—O1—N2	108.68 (17)	C10—C8—C7	111.13 (19)
C1—N1—C11	120.72 (17)	C8—C9—O1	109.8 (2)
C1—N1—C7	119.44 (18)	C8—C9—H9	125.1
C11—N1—C7	114.75 (16)	O1—C9—H9	125.1
C10—N2—O1	103.03 (18)	N2—C10—C8	114.6 (2)
N1—C1—C6	120.6 (2)	N2—C10—C11	133.3 (2)
N1—C1—C2	121.8 (2)	C8—C10—C11	112.02 (19)
C6—C1—C2	117.59 (19)	N1—C11—C10	100.43 (17)
C3—C2—C1	121.0 (2)	N1—C11—C12	113.51 (18)
C3—C2—H2	119.5	C10—C11—C12	114.16 (17)
C1—C2—H2	119.5	N1—C11—H11	109.5
C4—C3—C2	120.0 (2)	C10—C11—H11	109.5
C4—C3—H3	120.0	C12—C11—H11	109.5
C2—C3—H3	120.0	C14—C12—C13	111.1 (2)
C3—C4—C5	120.6 (2)	C14—C12—C11	112.28 (19)
C3—C4—Cl1	120.21 (18)	C13—C12—C11	110.13 (19)
C5—C4—Cl1	119.21 (18)	C14—C12—H12	107.7
C4—C5—C6	119.7 (2)	C13—C12—H12	107.7
C4—C5—H5	120.1	C11—C12—H12	107.7
C6—C5—H5	120.1	C12—C13—H13A	109.5
C5—C6—C1	121.0 (2)	C12—C13—H13B	109.5
C5—C6—H6	119.5	H13A—C13—H13B	109.5
C1—C6—H6	119.5	C12—C13—H13C	109.5
N1—C7—C8	101.51 (17)	H13A—C13—H13C	109.5
N1—C7—H7A	111.5	H13B—C13—H13C	109.5
C8—C7—H7A	111.5	C12—C14—H14A	109.5
N1—C7—H7B	111.5	C12—C14—H14B	109.5
C8—C7—H7B	111.5	H14A—C14—H14B	109.5
H7A—C7—H7B	109.3	C12—C14—H14C	109.5
C9—C8—C10	103.8 (2)	H14A—C14—H14C	109.5
C9—C8—C7	145.0 (2)	H14B—C14—H14C	109.5
			
C9—O1—N2—C10	0.3 (2)	C7—C8—C9—O1	175.2 (3)
C11—N1—C1—C6	170.09 (19)	N2—O1—C9—C8	0.1 (3)
C7—N1—C1—C6	16.6 (3)	O1—N2—C10—C8	−0.5 (3)
C11—N1—C1—C2	−9.7 (3)	O1—N2—C10—C11	177.8 (2)
C7—N1—C1—C2	−163.2 (2)	C9—C8—C10—N2	0.6 (3)
N1—C1—C2—C3	176.2 (2)	C7—C8—C10—N2	−176.7 (2)
C6—C1—C2—C3	−3.7 (3)	C9—C8—C10—C11	−178.10 (19)
C1—C2—C3—C4	1.0 (3)	C7—C8—C10—C11	4.6 (3)
C2—C3—C4—C5	2.2 (3)	C1—N1—C11—C10	−153.36 (19)
C2—C3—C4—Cl1	−175.91 (18)	C7—N1—C11—C10	1.3 (2)
C3—C4—C5—C6	−2.7 (3)	C1—N1—C11—C12	84.4 (2)
Cl1—C4—C5—C6	175.43 (17)	C7—N1—C11—C12	−121.0 (2)
C4—C5—C6—C1	0.0 (3)	N2—C10—C11—N1	178.2 (2)
N1—C1—C6—C5	−176.7 (2)	C8—C10—C11—N1	−3.5 (2)
C2—C1—C6—C5	3.2 (3)	N2—C10—C11—C12	−60.0 (3)
C1—N1—C7—C8	156.14 (19)	C8—C10—C11—C12	118.3 (2)
C11—N1—C7—C8	1.2 (2)	N1—C11—C12—C14	65.4 (2)
N1—C7—C8—C9	−178.9 (3)	C10—C11—C12—C14	−48.9 (3)
N1—C7—C8—C10	−3.4 (2)	N1—C11—C12—C13	−170.28 (18)
C10—C8—C9—O1	−0.4 (3)	C10—C11—C12—C13	75.4 (2)

### Hydrogen-bond geometry (Å, °)

**Table:**

Cg1 is the centroid of the C1–C6 ring.

**Table:**

*D*—H···*A*	*D*—H	H···*A*	*D*···*A*	*D*—H···*A*
C5—H5···Cg1^i^	0.95	2.65	3.405 (3)	136
C9—H9···Cg1^ii^	0.95	2.62	3.392 (3)	138

Symmetry codes: (i) −*x*+1, *y*−1/2, −*z*+1/2; (ii) *x*, −*y*+3/2, *z*+1/2.
